# Correction: Spatial-temporal patterns and predictors of timing and inadequate antenatal care utilization in Zambia: A Generalized Linear Mixed Model (GLMM) investigation from 1992 to 2018

**DOI:** 10.1371/journal.pgph.0005020

**Published:** 2025-08-05

**Authors:** Samson Shumba, Isaac Fwemba, Violet Kaymba

The third author’s name is spelled incorrectly. The correct name is: Violet Kayamba. The correct citation is: Shumba S, Fwemba I, Kayamba V (2024) Spatial-temporal patterns and predictors of timing and inadequate antenatal care utilization in Zambia: A Generalized Linear Mixed Model (GLMM) investigation from 1992 to 2018. PLOS Glob Public Health 4(10): e0003213. https://doi.org/10.1371/journal.pgph.0003213

## References

[1] Shumba S, Fwemba I, Kaymba V. Spatial-temporal patterns and predictors of timing and inadequate antenatal care utilization in Zambia: a Generalized Linear Mixed Model (GLMM) investigation from 1992 to 2018. PLOS Glob Public Health. 2024;4(10):e0003213. doi: 10.1371/journal.pgph.0003213 39471196 PMC11521255

