# Supplementary material for: Spatial-temporal patterns and predictors of timing and inadequate antenatal care utilization in Zambia: A Generalized Linear Mixed Model (GLMM) investigation from 1992 to 2018
Source: PLOS Glob Public Health. 2024 Oct 29;4(10):e0003213. doi: 10.1371/journal.pgph.0003213 (PMC11521255; doi:10.1371/journal.pgph.0003213)
Supplement: S1 Table — (DOCX) [file pgph.0003213.s001.docx]

| **Variables** | **1992** | **1996** | **Feb-01** | **2007** | **2013/14** | **2018** | **All years** |
| --- | --- | --- | --- | --- | --- | --- | --- |
|  | **(n=3,718)** | **(n=4,369)** | **(n=4,197)** | **(n=4,083)** | **(n=9,156)** | **(n=7,233)** | **(n=28,985)** |
| Region | <0.0001* | 0.0055* | -- | 0.2294 | <0.0001* | <0.0001* | <0.0001* |
| Central | 281 (85.88) | 322 (92.02) | 256 (86.35) | 336 (85.95) | 686 (80.53) | 473 (75.51) | 2354 (82.81) |
| Copperbelt | 826 (91.80) | 759 (89.66) | 620 (84.15) | 471 (79.26) | 937 (73.46) | 661 (68.61) | 4274 (80.39) |
| Eastern | 387 (94.06) | 588 (89.85) | 497 (87.91) | 494 (79.76) | 756 (64.16) | 497 (50.86) | 3219 (73.06) |
| Luapula | 211 (93.46) | 344 (88.46) | 497 (81.13) | 259 (77.22) | 536 (71.98) | 353 (55.60) | 1989 (74.16) |
| Lusaka | 566 (90.93) | 616 (88.31) | 461 (79.69) | 402 (77.71) | 1142 (75.83) | 846 (70.34) | 4034 (78.70) |
| Muchinga | -- | -- | -- | -- | 405 (76.05) | 215 (50.59) | 2355 (79.86) |
| Northern | 274 (91.16) | 432 (91.13) | 549 (90.75) | 480 (78.61) | 638 (81.80) | 375 (62.04) | 1610 (76.86) |
| North-western | 81 (86.93) | 144 (82.29) | 179 (84.76) | 192 (83.56) | 341 (78.31) | 265 (67.02) | 2280 (80.18) |
| Southern | 538 (85.28) | 390 (82.55) | 395 (83.66) | 351 (80.25) | 936 (74.56) | 562 (59.93) | 2517 (74.40) |
| Western | 169 (81.19) | 274 (88.32) | 326 (86.06) | 251 (85.46) | 500 (83.90) | 294 (63.32) | 795 (74.88) |
