# Supplementary material for: Spatial-temporal patterns and predictors of timing and inadequate antenatal care utilization in Zambia: A Generalized Linear Mixed Model (GLMM) investigation from 1992 to 2018
Source: PLOS Glob Public Health. 2024 Oct 29;4(10):e0003213. doi: 10.1371/journal.pgph.0003213 (PMC11521255; doi:10.1371/journal.pgph.0003213)
Supplement: Attachment — Submitted filename: Response to Reviewers.docx [file pgph.0003213.s002.docx]

The Editor,

PLOS Global Public Health

17^th^ August, 2024.

Dear sir/madam,

**Subject: Response to Reviewer Comments on Manuscript ID PGPH-D-24-00899**

I am writing to address the concerns raised by the reviewers regarding our manuscript entitled “**Spatial-Temporal Patterns and Predictors of Timing and Inadequate Antenatal Care Utilization in Zambia: A Generalized Linear Mixed Model (GLMM) Investigation from 1992 to 2018**” submitted to PLOS Global Public Health. We appreciate the constructive feedback provided, and we have made all necessary revisions to improve the quality of the manuscript.

The following is a summary of how we have addressed each concern:

**Reviewer 1**

1. Explain the rationale for choosing the GLMM model in your analysis. Describe why this model is appropriate for your data and research objectives

**ANS: When using the Demographic Health Survey (DHS) dataset, we opted for a generalized linear mixed-effects model (GLMM) with a logit link function over a mixed-effects logistic regression model due to the superior flexibility and robustness of GLMMs in handling the intricate hierarchical structure of DHS data. The GLMM approach allows us to incorporate both fixed effects and multiple levels of random effects, which is critical for accurately capturing the nested nature of the data that is, individuals within households, and households within enumeration areas. This method ensures that we properly account for the variability at each level, leading to more precise and reliable estimates of the predictors of health outcomes. By using GLMM, we can also effectively manage the complex survey design elements, such as stratification and clustering, which are pivotal in producing valid and generalizable inferences from the DHS dataset. This makes the GLMM a more appropriate and powerful tool for our analysis, enhancing the validity and reliability of our findings.**

2. Provide a clearer explanation of the model. Include the model equation, specifying which variables or levels were considered as random effects in your GLMM model. This will aid in understanding your analytical approach.

**ANS: The explanation has been provided, and the model equation has been entered. (page 6)**

3. Clarify whether enumeration areas (EAs) were considered as random effects in your model. If survey weights were used in the regression, mention this explicitly. If not, provide a justification for their exclusion.

**ANS: The EAs was considered as random effects in our model. Survey weights were not used in the regression mode because Multilevel models, by their nature, account for the hierarchical structure of the data, which helps address some aspects of survey design, such as clustering. This hierarchical modelling approach can provide robust estimates even in the absence of survey weights, as it explicitly models the nested data structure. (page 6)**

4. Given the extensive study period from 1992 to 2018, address potential variations by including the survey period as a random effect in your model. This adjustment can account for changes over time instead of considering the study period as a predictor variable.

**ANS: It has been incorporated as one of the random effects. (page 7)**

5. In Table 3, explain why data from 1992 to 2001-2 were excluded from the multivariate model, and only data from 2007 to 2018 were used. Provide a rationale for this decision.

**ANS: Since we have incorporated "year when the ZDHS was collected" as a random effect, we have removed it as a fixed effect variable.**

6. In the results section under "Quality of antenatal care offered between 2007 and 2018," you mention a significant increase in HIV testing rates from 46% to 98%.

Specify the statistical test used to determine this significance and provide the corresponding p-value.

**ANS: It has been corrected; the word "significant" has been removed as it was a typo. (page 20)**

**Minor comments**

1. Improve the quality of figures (Figure 1, Figure 2, Figure 3, and Figure 4) by uploading high-resolution images. Enhance the readability of legends, particularly in Figure 4.

**ANS: The figures have been separated to preserve the quality of the pictures**

2. Add details of each survey date and the number of women interviewed in Figure 1. Since different study period survey data were pooled, providing all this information in a single figure will make it more self-explanatory.

**ANS: Details added. The figure has been redone.**

3. Consider using a line chart instead of a bar chart to visually represent trends over time more effectively. Experiment with this change to see if it improves visual clarity.

**ANS: A line chart has been inserted for one of the figures, while we chose to retain bar charts for the other figures, as they more effectively convey the information.**

4. Ensure that tables are ordered sequentially. There is a duplication of Table 3; rectify this by renaming or renumbering as necessary.

**ANS: Correction made.**

5. Make the Table 3 (second one) self-explanatory. Ensure proper alignment of categories so that each variable is distinguishable from others.

**ANS: Corrected made. (page 16)**

6. In Table 2, Table 3, and Supplementary Table 1, ensure that individual "n" values add up to the total "n" for all years. Currently, the sample sizes do not match when pooling all survey data.

**ANS: The response variable was categorized as inadequate antenatal care (fewer than 8 sessions) and adequate antenatal care (8 or more sessions). In our frequency calculations, we only included instances of inadequate ANC in the table, with the remaining cases representing adequate ANC not being presented. This explains the discrepancy in the data. However, they adding up.**

7. Clarify why the study extracted pertinent variables only from the women's data files (individual recode) in the 2018 ZDHS dataset. Explain why data from other study years were not included.

**ANS: Corrected, should be from 1992 to 2018. (page 5)**

**Reviewer 2:**

Comments on

Title
1. The title is vague (not self -explanatory): Better to re-write in a way that clearly indicates the outcome of interest (inadequate ANC utilization)

**ANS: Title has been changed. (page 1)**

2. The paper analyses temporal trend and special distribution of number of ANC visits but the title doesn’t indicate that

**ANS: Title has been changed. (page 1)**

Abstract
1. The aim of the study is a mix-up of number of visit, timing of ANC, predictors, geospatial analysis, utilization, needs revision.

**ANS: It’s has been reconstructed. (page 4)**

Background
1. The first sentence is far from the aim of the study. Better to remove

**ANS: The sentence has been removed, and a more relevant statement has been added. (page 3)**

2. Line 7-11 of 1st paragraph confuses readers by merging causes of maternal and new-born mortality. Better to split the sentence

**ANS: Statement has been reconstructed. (page 3)**

3. 2nd paragraph-n sentences need bracket and full stops.

**ANS: Correction has been made. (page 3)**

4. Line 8 of 3rd paragraph doesn’t indicate which initiation rate: timely, global?

**ANS: Statement reconstructed. (page 3)**

5. Lastly, what was the knowledge gap which justifies the originality/conduct of this study?

**ANS: This paragraph on knowledge gaps has been added to the text which justifies the originality/conduct of this study. (see page 28)**

Methods
1. Data analysis section:

a. It would have been good if the model will Poisson for the number of ANC visits (count data)

**ANS: We aimed to compare inadequate antenatal care (fewer than 8 sessions) with adequate antenatal care (8 or more sessions), which is why using a Poisson model would not adequately address our research questions.**

b. Or the category should align with the policy recommendations as before 2016 (or the country adopting time) and after to say ANC is underutilized.

**ANS: We created this binary variable from the policy recommendation that was initiated in 2016, thus ANC sessions below 8 were inadequate compared to those 8 or more.**

c. Is it justifiable to use chi-square test over regression model

**ANS: We used a chi-square test for the baseline characteristics and applied a Generalized Linear Mixed Model (GLMM) regression to investigate the predictors.**

d. Better to come up with proper model fitness tests for categorized GLMM model.

**ANS: We proposed the log-likelihood ratio test, Akaike Information Criterion (AIC), and Bayesian Information Criterion (BIC) as model fitness tests. We believe these are reliable and consistent methods for determining the best-fit model. (page 8)**

e. On model selection section: Better to remove statistical jargon table 2 (diagnostic tests) for non-statistician readers and indicate your ICC value to see the warrantee for multilevel regression model.

**ANS: Noted it’s been removed. (See page 7 and 8)**

Result
1. Quality of ANC … In my opinion those are the components of ANC visits which are believed to be increased when number of visits improved. These my not reflect the “quality of care” so, better to state as ANC contents

**ANS: The statement has been corrected. (see page 18)**

Discussion
1. Paragraph 6, reveals there is special variation of ANC visit and speculated that the variation might be due to factors as economic, employment, etc.

a. Is it not possible to include those factors for the predictor analysis?

**ANS: The variables needed to validate this speculation, such as geographical economic activities and employment levels, are not captured in the ZDHS. Therefore, without these variables, we can only suggest possible effects, indicating a need for further investigation.**

b. Could you recommend further study to investigate the effects of those variables

**ANS: Yes, we do recommend more investigations in this area.**

c. Did you consider geographically weighted regression method for such ecological data?

**ANS: We chose to use a Generalized Linear Mixed Model (GLMM) instead of Geographically Weighted Regression (GWR) for analysing the Zambia Demographic Health Survey (ZDHS) dataset due to its hierarchical structure, with individuals nested within households and communities. GLMM effectively accounts for random effects and provides robust estimates of fixed effects, making it more suitable for understanding the complex relationships in the data while controlling for clustering, whereas Geographical Weighted Regression is not designed to handle such hierarchical data effectively. (see page 6)**

We believe these revisions have strengthened our manuscript, and we are confident that it now meets the high standards of PLOS Global Public Health. Enclosed with this letter, please find the revised manuscript along with a detailed point-by-point response to the reviewers’ comments.

Thank you for your consideration. We look forward to your favorable review and are hopeful for a positive outcome.

Yours sincerely,

Samson Shumba.
